# Transactions of the American Medical Association

**Published:** 1850-04

**Authors:** 


*Transactions of the American Medical Association.*
Vol. II. pp. 956.
(Concluded.)

REPORT ON MEDICAL EDUCATION.

This able report is, we presume, from the pen of Dr. F. Campbell Stewart, of New York, and embraces a discussion of the following topics:

Sec. 1.—An examination into the general condition of Medical Education in the United States, in comparison with the state of medical education in other enlightened nations; noticing, as occasion may call for, the courses of instruction, the practical requirements for graduation, the modes of examination for conferring degrees, and the number of pupils and of graduates at the several Medical Institutions in the United States.

Sect. 2.—The requirements of the United States Army and Navy Boards of Medical Examiners.

Sect. 3.—The legal requirements, exacted of medical practitioners in the several States of the Union.

Sect. 4.—Such measures, prospective or established, in reference to Medical Education and the reputable standing of the profession, as may be deemed worthy of special consideration.

To these will be added a fifth section, to comprise the consideration of certain matters not contemplated by the constitution, and which were specially referred to this committee.

It is our purpose to consider each of these several subjects separately, and we shall do this in the order in which they have been arranged.

Without following the committee through the first three sections, which may be considered as preliminary to the fourth, or to that which considers "such measures prospective or established in relation to medical education, and the reputable standing of the profession, as are deemed worthy of special consideration," we shall devote the few remarks we have to make on this report to this section.

It is to this branch of the question, that the minds of the profession are chiefly directed. The necessity of a progressive reform in our systems of medical education, is, we think, as deeply felt by the great mass of the intelligent and thinking portion of the medical men of the United States, as it was at the commencement of the movement, which resulted in the institution of the National

Medical Association. It is in fact to a wide spread conviction of growing errors and defects in the management of medical colleges, that the Association owes its existence; and the most prominent topic which occupied the attention of the first convention in New York, was that of medical education, in connection with the facility of obtaining diplomas, from corporations existing independent of the medical community, and irresponsible to it.

Two methods of correcting these evils suggested themselves to the friends of reform; one was, to seek the improvement of existing institutions, by certain recommendatory resolutions, and thus to elevate the standard of medical education, and render the diploma more valuable to its possessor; and the other, for the profession to take into their own hands the power of granting degrees, independent of medical colleges, and to receive into their ranks only such as had submitted to an examination by a Board of Examiners, who received their authority from the suffrages of their medical brethren, organized into State or County Medical Societies.

The former plan was deemed most advisable. It was argued, that recommendations coming from the assembled wisdom and intelligence of the profession, having for their object the improvement of medical education, would possess a weight and authority, which the colleges could not fail to respect, and that the professors in these institutions, some of whom were amongst the most eminent medical practitioners of the country, would cheerfully co-operate in any measures which might promote this desirable object, irrespective of their private interests.

The principal measures, recommended by the Association for this end, were:—

Firstly. The requirement of a more thorough preliminary education, from young men about to engage in the study of medicine, than has heretofore been requisite.

Secondly. The extension of the lecture term from four to six months.

Thirdly. A uniform and elevated standard of requirements for the degree.

Fourthly. The devotion of more time to clinical lectures, and study at the bed side.

Fifthly. More attention to practical anatomy and practical pharmacy.

To report how far these recommendations have been complied with, and thus to present an index of the progress of medical improvement, constituted the chief motive for the appointment of a permanent committee on Medical Education.

In regard to preliminary education, the committee believe that, owing to the rivalry of medical colleges, the facilities granted for obtaining diplomas, together with a want of a feasible plan for the accomplishment of the desired end, it has come to be entirely disregarded. To remedy this glaring defect, the committee propose the institution of "Primary Boards of Examiners," as recommended by the Medical Society of the State of New York.

These Boards are to be created by state and local medical societies, and their certificates of the qualifications of students to enter upon the study of medicine shall be required prior to their reception into the offices of practitioners or teachers.

In regard to the extension of the lecture term the committee remark:

"It affords us pleasure to inform you that the recommendations of the Philadelphia Convention, in regard to the *lengthening of the lecture term*, have been responded to by several of the most important and flourishing among the colleges; many of which have not only added to the length of the regular course, but given in addition a preliminary and gratuitous series of lectures.

The advantages arising from this improvement in our system are evident, and we are most happy to find that the apprehensions of those who feared that students would not consent to an increased tax upon their time, have not been fulfilled. The classes recently in attendance at those colleges which have extended the term, bear the same proportion to those of the colleges that have made no change as they formerly did, and in some instances they have been larger than ever. While we record the fact of the partial success of this innovation, and announce to you our belief that it meets with the approval of a large majority of the profession throughout the country, we regret to learn that, at one or two institutions, the enlightened Faculties of which were among the first to adopt the recommendation, a return to the old period of four months has been decided upon. This circumstance we regret the more, because we conceive that the period of one session was scarcely sufficient to test the matter fully, and because it may occasion hesitation and delay in other bodies that were about to make the change.

The attendance by students upon a term of six months is a reasonable exaction; it is only two-thirds of the time required *everywhere else than in this country;* and experience has shown that it is impossible for professors to impart the instruction that they are required to give, in a shorter period, without crowding and condensing to an extent which precludes the possibility of satisfactory and comprehensive illustration.

We feel convinced that it must ultimately prevail, and all that is wanting to insure its immediate and general adoption, is concert of action on the part of the colleges. We are satisfied that objections will not be raised by the student, when he is made fairly to understand that the change is principally for his advantage and convenience."

Upon the subject of elevating the standard of qualifications for a degree the committee are equally explicit.

"The importance of imposing additional restrictions upon students and practitioners, and of elevating, as far as practicable, the standard of education, should be admitted by all who are at all familiar with the subject.

It has been shown that, during the last five years, there have been graduated, annually, as many as from one thousand to fourteen hundred, by the colleges whose returns have been given, alone; and it is computed that as many as sixteen hundred diplomas are granted every year by the medical schools of the country. The number of candidates rejected is so small as scarcely to bear an appreciable proportion; and it is well known that not only many of the unsuccessful applicants for degrees, but also many of the students who have attended only one course of lectures, and never applied for an examination, enter at once upon the active duties of the profession, and engage in practice.

These are recognized and held by the public in the same light as regular graduates. The consequence is, that their want of general and professional knowledge, which is often manifested in courts of justice and elsewhere, reflects directly upon the profession of which they are supposed to be *bona fide* members, and tends materially to bring it into general disrepute. Nor is this want of a proper education confined exclusively to those who are possessed of no diploma.

We regret that candor and justice compel us to admit, that the most deplorable ignorance and unfitness for the responsible duties of a physician are often exhibited by those who hold the diploma, and who are thus endorsed as being qualified and competent. This fact is so notorious, that it would be a waste of time to cite instances in support of it. We may, however, assert, upon our personal responsibility, that we have known more than one graduate of some of our colleges, who could neither translate the Latin of his diploma, nor yet write and speak his own language correctly! The records of the army and navy show us how small a proportion of candidates for medical officers pass a satisfactory examination and are approved; and yet, nearly all of these are graduates in medicine, for the most part fresh from their studies, and subjected, not to a rigorous, but a fair and impartial examination."

The deficiency of hospital clinical instruction in our schemes of medical education, and the substitution in some of the medical colleges of what are termed "college clinics," for bed side instruction in hospitals, are condemned by the committee in most unqualified terms.

We were scarcely prepared for the assertion made by the committee, that "there are, comparatively speaking, but very few institutions in the country, whose rules require attendance upon bed side practice prior to graduation."

This deficiency the committee attribute to two causes.

"The first, is the circumstance of its not being generally insisted upon, and made obligatory; and the second, because so many subjects are taught in a very limited period of time, and so many lectures to be attended to, and prepared for, that the young men have, in reality, no time to devote to additional duties."

Upon the subject of college clinics the committee hold the following language:

"An attempt has been made within the few last years to afford a substitute for hospital attendance, by introducing what are called "College Clinics," and thus enabling professors to exhibit and explain to their students such cases of disease as may be presented for out-door treatment. That this system is better than no clinical instruction at all, we freely admit; but that it should ever be permitted to take the place of hospital attendance, when the latter can be procured, we contend to be wholly wrong. It is impossible for students to derive that advantage which is expected to result from witnessing the consecutive treatment of cases, by seeing persons affected with disease once or twice only, without being able to watch the effects of the medicinal agents employed, and the final result. There is another objection, likewise, to this imperfect system of clinical teaching which is worthy of consideration, as it affects the whole profession, and particularly those members of it whose practice is confined principally to the middle and lower classes of society, and whose business lies chiefly with that class of the community which is unable to pay a full compensation for medical services, and yet can easily afford some remuneration to their medical attendants. It is known to your committee that numerous individuals so situated, and well to do in the world, are in the habit of resorting to the cliniques of the schools to obtain, gratuitously, advice for which they are fully able to pay. Thus a direct and positive injury is inflicted upon a large and deserving class of our brethren, whose means of livelihood and pecuniary receipts are proportionally curtailed.

We concur unanimously in the opinion that they present no adequate equivalent to the student, when resorted to as a substitute for actual hospital attendance, while, at the same time, they are a direct source of injury to many deserving members of the profession."

In regard to the study of practical anatomy, we are informed by the committee that a most glaring deficiency exists in some of our medical colleges, which even announce in their published circulars,

"that attention to it will not be made obligatory upon those who enrol themselves as their pupils."

Attention to practical pharmacy is equally disregarded, though this is often taught in the offices of country practitioners, where the preceptor requires the aid of his pupils in preparing and putting up medicines.

The committee justly regard atttention to this department of medicine, of the greatest importance, and recommend the course adopted in Edinburgh as worthy of imitation; viz., "that prior to receiving his diploma, every candidate should be required to produce evidence of his having been engaged, during a period of at least three months, in compounding medicines, either in his preceptor's office, or in the shops of recognized apothecaries, members of a college of pharmarcy."

In the admirable scheme of private instruction devised by the late Dr. Parrish, under whose guidance some of the most distinguished medical men of the country commenced their studies, *six* months in the shop of a scientific apothecary was regarded as an essential preliminary to a thorough medical education.

The last subject discussed by the committee, is "the propriety of establishing independent Boards of Examiners, for conferring licenses to engage in the active practice of medicine and surgery." In this matter the committee concur in the opinion, that the profession require protection, and they regard legal measures as the only effectual means of securing it.

The following extract from the report exhibits some of the grounds upon which this conclusion is based:

"It appears to us that there are but two courses to be pursued to insure the public and the profession that protection which both are entitled to claim and expect: the one is through the profession itself, and the schools; and the other through the law.

So long as charters for medical colleges can be obtained with the facility which is now afforded, and so long as professorships continue to be sought after, not only for the direct profits which they yield, but also for the indirect and valuable advantage which they offer by enabling those who hold them to become extensively and favourably known, thus leading to the attainment of business and distinction, every inducement will be held out to young men to engage in the study of the profession—and rivalry and competition among the institutions themselves, to obtain popularity and large classes, must lead inevitably to the cheapening of the degree, and a lessening of the requirements for obtaining it. We all know that the reputation and success of an institution are proportionate with the size of its classes, and the number of its graduates. Hence, every means is resorted to for obtaining the foremost numerical position. The degrees from all the colleges confer the same rights, and yield similar honours: the public look only at the parchment, and make no distinction as to the source whence it was obtained. This being the case, no inducement is held out to the teachers to impose adequate restrictions upon their pupils, and none is offered to the pupils themselves for incurring more trouble and expense than may be absolutely necessary for obtaining the doctorate.

We think that no material and lasting good can arise from an elevation of the standard of requirements by one or two colleges alone. The only manner in which the desired end could be obtained would be, by the coöperation of all the colleges, in the establishment of a uniform curriculum;—and is this practicable? Can it be expected that rival institutions, possessing a great diversity of interests, and unequal facilities for teaching, will unite in a general scheme, which would have the effect of concentrating students at such colleges as are in reality best prepared to impart instruction? We unhesitatingly answer in the negative—it is useless even to ask it. But let each State of our Union authorise the establishment of a board of disinterested examiners, who shall have no direct interest in view, and the obtaining of whose certificate shall be always obligatory, prior to permitting an individual to engage in practice, and a direct check is at once imposed, which will make it to the interest of the schools to raise their standard to the level of that established by the licensing boards, and a fair and just rivalry will then commence between the colleges, as to which shall impart the requisite amount of information in the shortest space of time, and at the least expense to the student."

How far the conclusions of the committee will be sustained by the future action of the Association, is yet to be proven. It must be confessed, that the measures, so auspiciously commenced for imparting to the schools a higher tone of instruction, and a deeper sense of their responsibility in granting the degree, have not, thus far, been as successful as the profession had reason to expect. To this fact the report before us, bears ample evidence. But few of the medical colleges have made any important changes in their curriculum of studies, while others have adopted regulations altogether at variance with the expressed sentiments of the Association. It is to be feared indeed that some of these institutions are disposed to act independently of the profession, and to pursue such course, both in the education of their students, and in the granting of the degree, as their own judgment or their supposed pecuniary interests may dictate. That such corporations have a right, under the laws of the land, to adopt this course, cannot be questioned, while the duty of the great body of the profession to protect themselves and the public against what they conceive to be an injurious and degrading policy, is equally clear and imperative.

The plan proposed by the committee, emanating as it does from gentlemen of high character, specially selected to examine into this important and difficult question, is at least entitled to the most respectful consideration from the body who intrusted to them this duty.

The committee have not failed to perceive the difficulties attendant upon the organization of independent boards of examiners, in the several States, invested by law with the power of granting the degree. They foresee impediments which may arise on the part of medical colleges which in many of the States now exclusively exercise this prerogative. But inasmuch as legal restrictions to the practice of medicine are rarely imposed, and as irregular practitioners without diplomas may and do practice with the same facility as those who hold them, it is suggested that the right to practice conferred by these charters is in fact of little practical value; and that the educated and high minded men who occupy the chairs in many of our medical colleges, would readily yield to the general voice, and abandon a valueless prerogative, especially if they could see that by so doing they would be advancing the general welfare.

Embarrassments might also arise in carrying out the details of the plan proposed by the committee, even admitting that the principle upon which it is based, is recognized by the state legislatures ; but these are not insurmountable if the question is seriously entertained, and if the united influence of the profession is brought to bear upon its accomplishment.

In the State of New Jersey, Examining Boards appointed by the State Medical Society have long been in successful action, and no physician can be admitted into medical fellowship in that state, or can be invested with a legal right to practice there, unless he holds a license from such a Board. The consequence is, that in no state in the Union, is the medical profession more respectable and better informed, and in none does quackery find less encouragement.

Appended to the report are two papers, one from the medical faculty of Harvard University in opposition to extension of the lecture term beyond four months, and the other in favor of this measure from a special committee appointed by the Association to sustain the course of action which it has uniformly recommended, as an important means of improving our systems of medical education.

The length to which our remarks have extended, will preclude an examination into the merits of these able papers; while we cannot withhold our regret that the medical faculty of one of the most renowned institutions of learning in the country, should be found arrayed against a measure which the general voice of the profession had so formally sanctioned: we fear that this course will encourage inferior schools in continuing their imperfect courses, and even in further curtailment, if they deem it important to their interests.

With these observations we must take leave of the volume of Transactions, without even a passing notice of the other and varied contents which enrich its pages. The vexatious delay which has so far prolonged its appearance, (for which the committee of publication are not responsible,) will we trust be avoided in future. In consequence of it, little time has been afforded for the perusal and study of the many valuable papers which the volume contains, by the great mass of those who are intimately interested in these proceedings. We trust, however, that the work will meet with the reception which it so justly merits, and that it will find a place on the shelves of all the thinking and active practitioners throughout the Union.

